# Rhigolene.—A Petroleum Naphtha for Producing Anæsthesia by Freezing

**Published:** 1866-09

**Authors:** Henry J. Bigelow

**Affiliations:** Professor of Surgery in the Massachusetts Medical College


### Selections.

### RHIGOLENE.—A PETROLEUM NAPHTA FOR PRODUCING ANÆSTHESIA BY FREEZING.

[Read before the Boston Society for Medical Improvement, April 9th 1866, and communicated for the Boston Medical and Surgical Journal.]

By Henry J. Bigelow, M. D., Professor of Surgery in the Massachusetts Medical College.

The above name is proposed as convenient to designate a petroleum naphtha boiling at 70° F., one of the most volatile liquids obtained by the distillation of petroleum, and which has been applied to the production of cold by evaporation. It is a hydrocarbon, wholly destitute of oxygen, and is the lightest of all known liquids, having a specific gravity of 0.625. It has been shown that petroleum, vaporized and carefully condensed at different temperatures, offers a regular series of products which present more material differences than that of their degree of volatility, and that the present product is probably a combination of some of the known products of petroleum with those volatile and gaseous ones not yet fully examined, and to which this fluid owes its great volatility. A few of these combinations are already known in trade, as benzolene, kerosene, kerosolene, gasolene, &c., all of them naphthas, but varying with different manufacturers. I procured, 1861, a quantity of kerosolene of four different densities, and found the lightest of them, the boiling point of which was about 90°, to be an efficient anæsthetic by inhalation. When it was learned here that Mr. Richardson, of London, had produced a useful anæsthesia by freezing through the agency of ether vapor, reducing the temperature to 6° below zero, F., it occurred to me that a very volatile product of petroleum might be more sure to congeal the tissues, besides being far less expensive, than ether. Mr. Merrill having, at my request, manufactured a liquid of which the boiling point was 70° F., it proved that the mercury was easily depressed by this agent to 19° below zero, and that the skin could be with certainty frozen hard in five or ten seconds. A lower temperature might doubtless be produced, were it not for the ice which surrounds the bulb of the thermometer. This result may be approximately effected by the common and familiar "spray producer," the concentric tubes of Mr. Richardson not being absolutely necessary to congeal the tissues with the rhigolene, as in his experiments with common ether. I have for convenience used a glass phial, through the cork of which passes a metal tube for the fluid, the air-tube being outside, and bent at its extremity so as to meet the fluid-tube at right angles, at some distance from the neck of the bottle. Air is not admitted to the bottle, as in Mr. Richardson's apparatus, the vapor of the rhigolene generated by the warmth of the hand applied externally being sufficient to prevent a vacuum and to ensure its free delivery; 15° below zero is easily produced by this apparatus. The bottle, when not in use, should be kept tightly corked, a precaution by no means superfluous, as the liquid readily loses its more volatile parts by evaporation, leaving a denser and consequently less efficient residue. In this, and in several more expensive forms of apparatus in metal, both with and without the concentric tubes, I have found the sizes of 72 and 78 of Stubs's steel wire gauge to work well for the air and fluid orifices respectively; and it may be added that metal points reduced to sharp edges are preferable to glass, which, by its non-conducting properties, allows the orifices to become obstructed by frozen aqueous vapor.

Freezing by rhigolene is far more sure than by ether, as suggested by Mr. Richardson, inasmuch as common ether, boiling only at about 96° instead of 70°, often fails to produce an adequate degree of cold. The rhigolene is more convenient and more easily controlled than the freezing mixtures hitherto employed. Being quick in its action, inexpensive and comparatively odorless, it will supersede general or local anæsthesia by ether or chloroform for small operations and in private houses. The opening of felons and other abscesses, the removal of small tumors, small incisions, excisions and evulsions, and perhaps the extraction of teeth, may be thus effected with admirable ease and certainty; and for these purposes surgeons will use it, as also, perhaps, for the relief of neuralgia, chronic rheumatism, &c., and as a styptic, and for the destruction by freezing of erectile and other growths. But for large operations it is obviously less convenient than general anæsthesia, and will never supersede it. Applied to the skin, a first degree of congelation is evanescent; if protracted longer, it is followed by redness and desquamation, which may be possibly averted by the local bleeding of an incision; but if continued or used on a large scale, the dangers of frost-bite and mortification must be imminent.

It may be superfluous to add that both the liquid and the vapor of rhigolene are highly inflammable.

